# The Impact of Different Hydrocolloids on the Viscoelastic Properties and Microstructure of Processed Cheese Manufactured without Emulsifying Salts in Relation to Storage Time

**DOI:** 10.3390/foods11223605

**Published:** 2022-11-12

**Authors:** Alena Kratochvílová, Richardos Nikolaos Salek, Martin Vašina, Eva Lorencová, Vendula Kůrová, Zuzana Lazárková, Jolana Dostálová, Jana Šenkýřová

**Affiliations:** 1 Department of Food Technology, Faculty of Technology, Tomas Bata University in Zlin, T.G. Masaryka 5555, 760 01 Zlin, Czech Republic; 2 Department of Hydromechanics and Hydraulic Equipment, Faculty of Mechanical Engineering, VŠB-Technical University of Ostrava, 17. listopadu 15/2172, 708 33 Ostrava, Czech Republic

**Keywords:** processed cheese, hydrocolloids, rheology, microstructure, storage time

## Abstract

The current study was conducted to evaluate the effect of the addition of selected hydrocolloids [agar (AG), κ-carrageenan (KC), or gelatin (PG); as a total replacement for emulsifying salts] on the viscoelastic properties and microstructure of processed cheese (PC) samples during a storage period of 60 days (at 6 ± 2 °C). In general, PC viscoelastic properties and microstructure were affected by the addition of hydrocolloids and the length of storage time. The evaluated PC reported a more elastic behavior (G′ > G″) over the viscous one. The highest values of viscoelastic moduli (G′; G″; G*) were recorded for PC samples manufactured with KC addition, followed by those prepared with AG and PG. The control sample presented values of viscoelastic moduli similar to those of the PG sample. All PC samples tested had fat globule size values lower than 1 μm. Moreover, PC with AG and KG addition presented similar microstructures and sizes of fat globules.

## 1. Introduction

In general, processed cheese (PC) is a dairy-based gel characterized as a stable oil-in-water emulsion with viscoelastic properties [1,2,3]. Furthermore, PC is a dairy product that differs from natural cheese because it is not manufactured directly from milk. The main ingredient in PC production is/are natural cheese/cheeses (of various types and degrees of maturity). The smooth and homogeneous consistency of PC is formed by mixing natural cheese with appropriate emulsifying salts (ES), heated under moderate pressure and constant shear, commonly at a temperature within the range of 90–100 °C [4,5]. Moreover, the development of the desired PC uniform consistency can be influenced by three groups of factors, including (i) composition and properties of the raw materials utilized (type and maturity level of the natural cheeses, pH of the natural cheese to be melt, dry matter (DM) and fat in dry matter (FDM) contents, ES type and/or concentration, possible use of hydrocolloids), (ii) processing conditions (stirring speed and duration, cooling time and rate) and (iii) storage conditions (storage time and temperature, packaging material properties) [5,6,7]. 

During the manufacture of PC, ES play a crucial role in the manufacture of PC, leading to the development of homogeneous products with the desired consistency. In particular, the key role of ES is Ca sequestration, leading to the development of the more soluble Na-paracaseinate, which in turn can act as an active emulsifier. Additionally, the application of ES results in emulsification and stabilization of the fat present in the PC matrix because protein chain peptization, dispersion, hydration, and swelling will occur [8,9]. Phosphates and citrates or combinations of these are most commonly used in the manufacture of PC and similar products [8,10].

From the point of view of human nutrition, an ideal ratio of Ca and P absorbed is 1:1. However, the aforementioned ratio in PC is generally reduced to 1:1.5–3.0 due to the presence of phosphate-based ES [11,12,13]. According to Černíková et al. [14], a possible decrease in the P amount in PC could result in its better nutritional rating. Replacement of the ES can be realized according to the following three approaches. The first option is to reduce the amount of ES by partially replacing them with other substances. The second variant is to substitute ES with other food additives or mixtures completely. Finally, the last approach consists of partially removing calcium ions from the raw material mixture by physical or physicochemical methods [14,15,16]. Moreover, the successful substitution of ES by hydrocolloids in PC allows for (i) a decrease of the level of P and an increase in the Ca:P ratio; (ii) a decrease of the Na concentration; (iii) utilization of biodegradable food additives from alternative sources instead of P; (iv) formation of PC products with potential health benefits for consumers. 

Hydrocolloids are a heterogenous group of long-chain biopolymers (polysaccharides and/or proteins) that are mainly applied in various food systems to create and disperse emulsions, stabilize foams, and form gels. The typical level of hydrocolloids added to food products is less than 1%. Furthermore, natural/modified starches, carrageenan, pectin, agar, xanthan gum, locust bean gum, guar gum, gum arabic, gelatin, casein, caseinates, and whey proteins are among the hydrocolloids most frequently applied in the food industry [13,17,18].

To our knowledge, no study has been found to date that provides a direct comparison of viscoelastic characteristics and microstructure of PC manufactured using agar (AG), κ-carrageenan (KC), or gelatin (PG) as a total replacement for ES. Therefore, the main objective of the current study was to evaluate the effect of different gel hydrocolloids (AG, KC, PG) addition (as a total replacement for ES) on the viscoelastic properties and microstructure of PC samples during a storage period of 60 days (at 6 ± 2 °C). Moreover, a secondary objective was to compare the developed viscoelastic properties and microstructure of PC with hydrocolloids in addition to that of a control sample (CS; PC in which conventional phosphate-based ES was used).

## 2. Materials and Methods

### 2.1. Materials

For the preparation of PC samples with a DM content of 40% *w/w* and an FDM content of 55% *w/w*, the following raw ingredients were used: Edam cheese (8-week maturity; 50% *w/w* DM, 30% *w/w* FDM; Kromilk, a.s., Kroměříž, Czech Republic); butter (82% *w/w* DM; Sachsenmilch Lepperdorf, GmbH, Wachau, Germany) and hydrocolloids: KC, AG, and PG (Sigma-Aldrich, Inc., Saint Louis, MO, USA). ES were purchased from Fosfa PLC Company (Břeclav, Czech Republic). Glutaraldehyde solution, cacodylate buffer, ethanol, and chloroform were obtained from Sigma-Aldrich, Inc. (Saint Louis, MO, USA).

### 2.2. Manufacture of Processed Cheese Samples

The model PC samples were manufactured using the Stephan UMC-5 (Stephan Machinery GmbH, Halmen, Germany) equipped with indirect heating. The target temperature was set at 90 °C with a holding time of 60 s, and the raw materials mixture was thermally treated under a partial vacuum. The stirring speed of 3000 rpm was applied. The PC samples were manufactured according to the production schema previously reported by Pluta-Kubica et al. [19]. The formulation of PC samples is presented in Table 1. The hot PC melt was filled in cuboid shape polypropylene containers (length: 95 mm, width: 75 mm, height: 30 mm). The filled containers were sealed with aluminum lids and allowed to cool. Subsequently, the PC samples were transferred to a refrigerator (at 6 ± 2 °C), where they were stored throughout the experiment. The analyses were realized on the 1st, 14th, 30th, and 60th days after the production day (day 0 was the production day).

### 2.3. Basic Chemical Analysis

The DM and fat contents of the evaluated PC samples were determined according to ISO 5534 [20] and ISO 1735 [21], respectively. The pH values were measured at 20 ± 1 °C using the glass tip electrode of a pH meter (pH Spear, Eutech Instruments Europe B.V., Landsmeer, The Netherlands) by directly inserting the spear into the PC samples in 3 randomly selected spots (in each packaging). The recorded values were the mean of nine repetitions (*n* = 9).

### 2.4. Rheological Measurements of Processed Cheese Samples

The determination of PC viscoelastic properties was performed using a dynamic oscillatory shear rheometer (RheoStress 1, Haake, Bremen, Germany) equipped with a parallel plate-plate geometry (diameter: 35 mm). Rheological measurements were made at 20.0 ± 0.1 °C, and a 1.0 mm gap was applied. In order to determine the linear viscoelastic regions (LVR), amplitude sweeps were performed. Therefore, the amplitude of shear stress of 20 Pa was selected within the LVR. The exposed edge of the parallel plate-plate geometry was covered with a thin layer of silicone oil to prevent the PC samples from dehydrating. During the tests, the elastic (G′; Pa) and viscous (G″; Pa)Pa) moduli were recorded in a frequency range of 0.01–100.00 Hz, and subsequently, the complex modulus of elasticity (G*; Pa) was calculated according to the following Equation (1):

(1) $$G^{*}=\sqrt{{\left(\;G\;\prime \right)}^{2}+{\left(\;G\;\text{″}\right)}^{2}}$$

The frequency of 1 Hz was chosen as the reference frequency for the presentation of the complex viscosity η, tan δ, and G*. The recorded values were the mean of nine replicates (*n* = 9).

### 2.5. Scanning Electron Microscopy of Processed Cheese Samples

The Jeol JSM-7401F scanning electron microscope (Jeol, Tokyo, Japan) was implemented to evaluate the microstructure of the model PC samples. PC samples were prepared according to Černíková et al. [22]. In particular, part of the PC sample (size = approximately 5 × 2 × 2; mm) was placed in a glutaraldehyde solution of 3% (*v*/*v*) in a cacodylate buffer of 0.2 mol/L for a 24 h period. The samples were then washed in cacodylate buffer (3 times for 15 min). Postfixation was performed for 48 h in 2% (*w*/*v*) OsO_4_. The samples were washed again in cacodylate buffer (3 times for 15 min). Furthermore, PC samples were dehydrated by solutions with an increased concentration of ethanol from 30 to 100%. The samples were subsequently frozen, fractured in liquid nitrogen, and defatted with chloroform. Fragments of the model PC were dried with carbon dioxide (Leica EM CPD300, Leica Microsystems, Vienna, Austria). The samples were mounted on an aluminum stem and holder and sputter coated (Sputter Coater SCD 050, Bal-Tec, Balzers, Liechtenstein) for a period of 98 s (20-nm layer of gold). The samples were viewed using a Jeol JSM-7401F (Jeol, Tokyo, Japan) scanning electron microscope, and each image was analyzed using ImageJ software (US National Institute of Health website, https://www.nih.gov/, accessed on 14 March 2022). The image of each PC sample was analyzed to determine the diameter of the fat globule (μm). Each PC sample was analyzed twice, and the results were expressed as the median ± standard deviation.

### 2.6. Statistical Analysis

The results represented the average means with standard deviation (SD) of repeated measurements (*n* = 9). Nonparametric statistical methods were used in this study. Two-factor Kruskal–Wallis analysis of variance (ANOVA) was applied to determine the effect of hydrocolloid type addition (factor A) and storage period (factor B) on the viscoelastic properties of PC. Multiple comparison procedure among means was performed using Tukey’s method. The chi-square test was applied to compare the fat droplet size of the model PC. All the statistical analysis methods were performed at the probability level of *p* ˂= 0.05 (Minitab, Ltd., Coventry, UK).

## 3. Results and Discussion

### 3.1. Basic Chemical Analysis of Processed Cheese Samples

**Figure 1:**
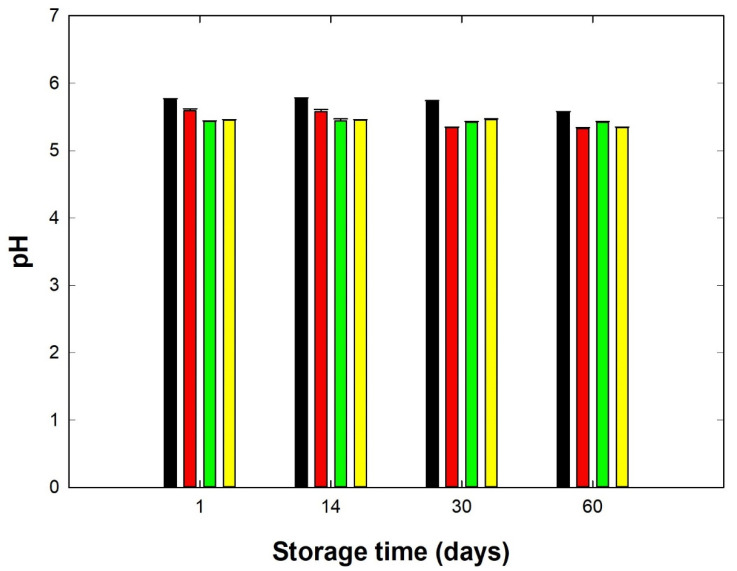
Development of pH values for processed cheese samples manufactured with different types of hydrocolloids: [control sample (black), agar (red), κ-carrageenan (green), and gelatin (yellow)] during a storage period of 60 days at 6 ± 2 °C [*n* = 9; the results were expressed as means (columns) and standard deviations (bars); processed cheese samples were sampled after 1, 14, 30 and 60 days of storage].

The DM content of the model PC samples ranged from 40.54 to 41.42% (*w/w*), providing valuable information on the stability of the DM content of the samples. Furthermore, the fat content of the PC samples was in the range of 22.5% to 23.0% (*w/w*). Furthermore, another significant factor affecting the functional and viscoelastic properties of PCs is the pH value. Figure 1 shows the development of the pH values of the PC samples as affected by the length of storage time. The pH values of the samples produced with the addition of hydrocolloids were within the 5.33–5.59 interval throughout the storage time (*p* < 0.05). Furthermore, the pH values of the CS were in the range of 5.57 to 5.77. However, during the 60-day storage period, a slight decrease in pH was observed in all samples tested (*p* < 0.05). According to Černíková et al. [22], a possible decrease in the pH values during storage by 0.1–0.2 can be explained by the hydrolysis of diphosphates and polyphosphates or by possible changes in the dissociation of ES or other compounds present in the developed PC-matrix. In terms of PC, in which hydrocolloids were added, there was no significant decrease (*p* ≥ 0.05) in pH values during storage. The pH results of the PC samples manufactured with the addition of AG as a total substitute for ES approached a pH value of 5.6 only on the first and fourteenth day of storage, and then the pH decreased to 5.3. For PC samples with the addition of KC, the pH values were nearly 5.4, and the pH values did not decrease further with increasing storage time. Furthermore, the pH value of the PC samples containing PG was 5.4 at the beginning of the experiment, and after 60 days of storage, the pH value decreased to 5.3.

### 3.2. Rheological Measurements

**Figure 2:**
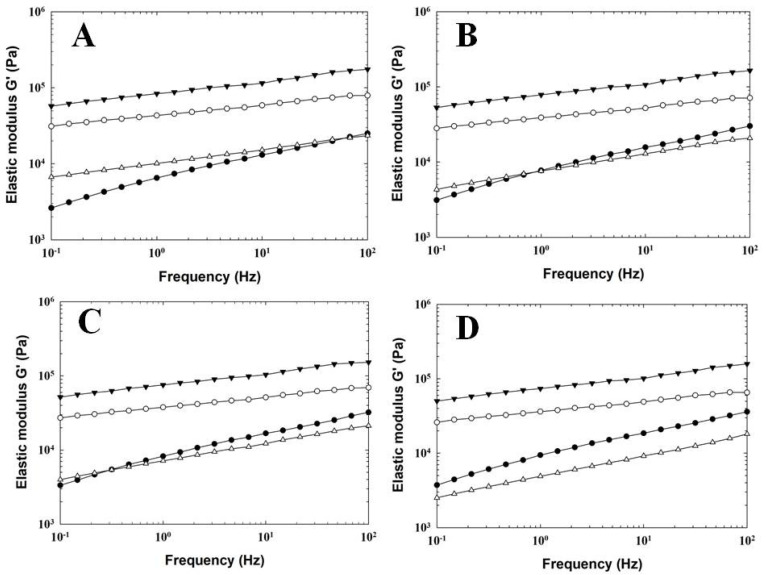
Development of elastic modulus G′ values for processed cheese samples manufactured with different types of hydrocolloids (control sample—full circle; agar—open circle; κ-carrageenan—full triangle; gelatin—open triangle;) without emulsifying salts, a storage period of 60 days (at 6 ± 2 °C). Processed cheese samples were sampled after 2 (part (**A**)), 9 (part (**B**)), 30 (part (**C**)), and 60 (part (**D**)) days of storage.

**Figure 3:**
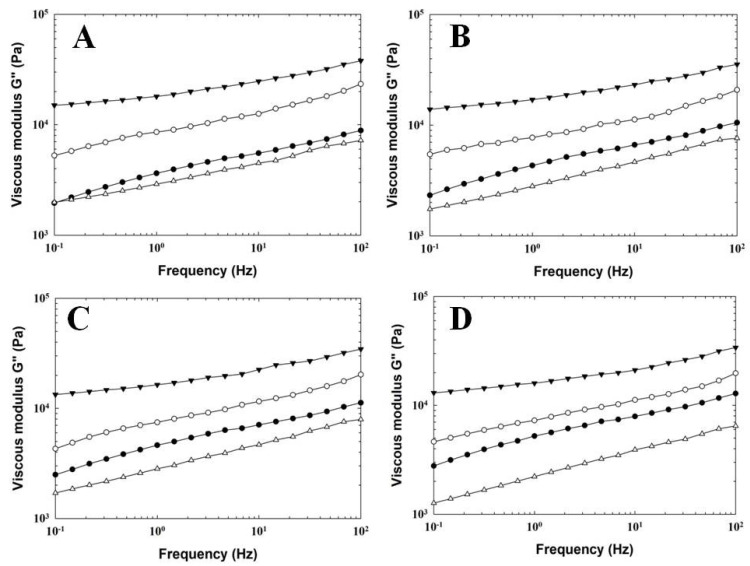
Development of viscous modulus G″ values for processed cheese samples manufactured with different types of hydrocolloids (control sample—full circle; agar—open circle; κ-carrageenan—full triangle; gelatin—open triangle) without emulsifying salts during a storage period of 60 days (at 6 ± 2 °C). Processed cheese samples were sampled after 2 (part (**A**)), 9 (part (**B**)), 30 (part (**C**)), and 60 (part (**D**)) days of storage.

Rheology can prove to be a very valuable tool that allows for direct insight into the functional properties (in terms of consistency) of a material [23]. The results of dynamic oscillatory rheometry for PC samples manufactured with the addition of three individual hydrocolloids (as a total replacement for ES) and CS are depicted in Figure 2 and Figure 3 and Table 2.

From the results obtained, it can be stated that higher values of elastic (G′) and viscous (G″) moduli were recorded for PC samples, in which KC and AG were used. In comparison, samples with PG addition showed lower values of both tested moduli (G′ and G″) compared to CS. Generally, all tested PC samples presented more elastic behavior (G′ > G″). According to the rheological definition of a gel, the latter is a viscoelastic system with an ‘elastic’ modulus (G′) larger than the ‘viscous’ modulus (G″). Furthermore, G′ is a measure of the energy that is elastically stored in the structure of the material (gel) during a periodic application of stress, and G′ is a measure of the energy dissipated (or the viscous response). The ratio equal to G″/G′ (i.e., tan δ) provides information about how much stress and strain are out of phase with each other [18,24]. The results obtained show that the application of three individual hydrocolloids influenced the viscoelastic properties of the model PC samples. The highest values of G′ (Figure 2) were recorded for PC samples manufactured with KC addition, followed by those prepared with AG and PG. The CS presented values of G′ similar to those of PG. The G″ values of the tested PC samples followed a similar trend, as was previously mentioned (Figure 3). Generally, with increasing frequency, a gradual increase in the values of both recorded moduli was observed for all investigated PC samples.

Table 2 presents the results of complex modulus of elasticity (G*), tan δ, and complex viscosity (ŋ) (for the reference frequency of 1 Hz) for PC samples manufactured with hydrocolloids as a function of storage time. The data provided shows that the sample with the addition of 1% (*w*/*w*) KC showed the highest G* values, followed by the PC sample, in which AG was used. On the other hand, the sample in which PG was added showed G* values similar to those of the CS. According to Piska and Štětina [25], an increase in the values of G* would result in elevated stiffness of PC. Černíková et al. [26] concluded that elevated concentration of KC could lead to changes in gel properties or to more intensive interactions between carrageenan chains, resulting in the formation of a “dense” network structure and, therefore, increasing the stiffness of the PC. Gels formed with KC addition present synergistic effects in terms of high values of moisture, elasticity, and viscosity. Furthermore, when the G* values of PC samples manufactured with AG and KC were compared, the sample with the addition of KC presented G* values almost two times higher than the sample in which AG was used. The latter phenomenon could be explained by the fact that AG is very weakly compared to neutrally charged polysaccharides and does not interact with proteins or other charged molecules, forms weak secondary and branched networks, and exhibits lower syneresis compared to anionic polysaccharides such as pectin, к-carrageenan, or xanthan gum [27,28,29].

In general, the samples prepared with the addition of gelatin presented low values of all tested viscoelastic moduli (G′; G″; G*), similar to those of CS. It is generally known that gelatin is proteinaceous in nature, has the ability to form a protein network, and binds to water, preventing syneresis [30]. Additionally, when comparing PC samples with the addition of PG and AG, it was found that samples containing gelatin had G* values of about a quarter lower compared to samples with AG addition. 

In CS produced with the addition of ES, a slight increase in G* was observed during the storage period (*p* ≥ 0.05). On the contrary, the rigidity of PC samples manufactured using hydrocolloids showed a significant (*p* < 0.05) decrease in G* over the whole experiment. Therefore, a hypothesis might be suggested that during storage, the structures of the hydrocolloid-protein complex are probably rearranged, and the present water is bound less intensively to the developed matrix. Furthermore, the aforementioned decrease in G* resulted in PC samples, probably due to the smaller number of interactions within the matrix occurring during storage [6]. The results of complex viscosity agreed with those of G*. 

### 3.3. Scanning Electron Microscopy

**Figure 4:**
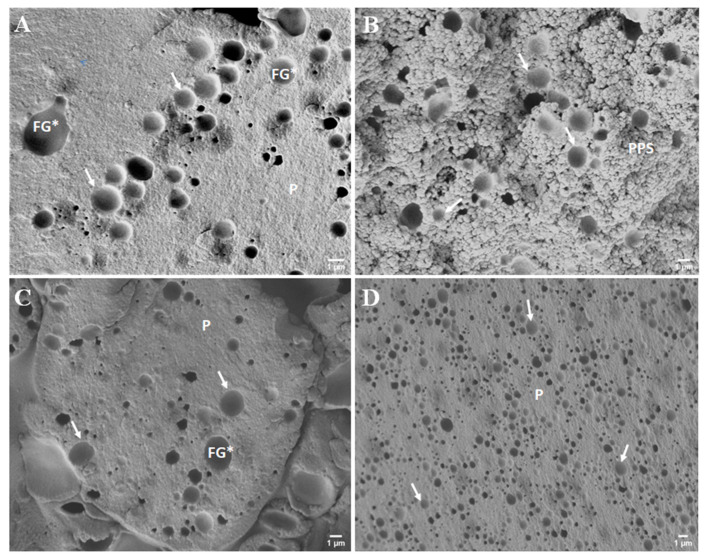
Scanning electron microscopy images of the model processed cheese (the scale bars represent 1 μm; 5000× magnification). (**A**) Sample with agar; (**B**) sample with κ-carrageenan; (**C**) sample with gelatin; (**D**) sample with emulsifying salts; FG* (and white arrows)—void space of fat globules; P—protein structure; PPS—protein-polysaccharide structure.

**Figure 5:**
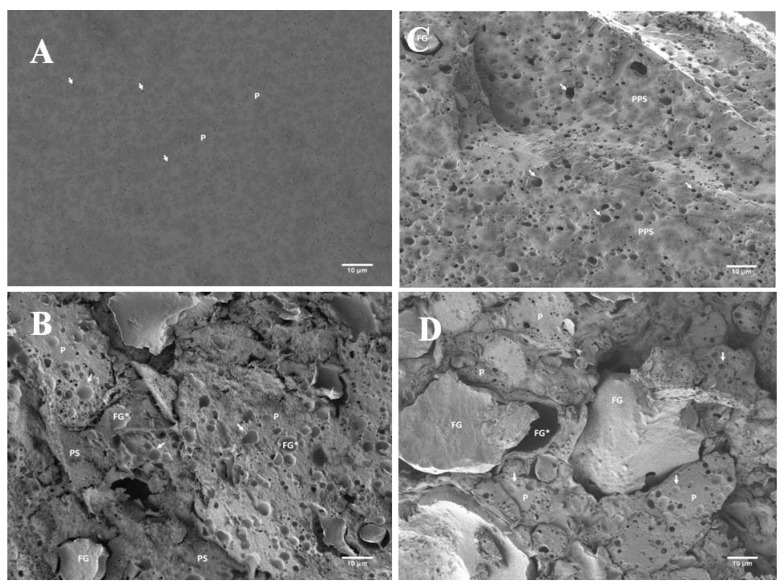
Scanning electron microscopy images of the model processed cheese (the scale bars represent 10 μm; 1000× magnification). (**A**) Sample with emulsifying salts; (**B**) sample with agar; (**C**) sample with κ-carrageenan; (**D**) sample with gelatin; FG* (and white arrows)—void space of fat globules; P—protein structure; PPS—protein-polysaccharide structure.

In addition to the interconnection of the developed PC matrix evaluated by the viscoelastic properties, the different consistencies of the evaluated PC with the addition of hydrocolloids can also be explained by the varying sizes of the fat droplets (Figure 4 and Figure 5 and Table 3). Statistically, the median value of the size of the fat droplets (Table 3) in CS with added ES differed significantly from the samples with added hydrocolloids (*p* < 0.05). The size of the fat globules of the samples was determined using scanning electron microscopy, and it was found that the samples with the addition of ES showed the lowest values (Table 3), indicating that the emulsification of the fat present within the PC matrix was much more effective than in the samples with the use of hydrocolloid. In general, all PC samples tested presented fat globule size values lower than 1 μm. Furthermore, ES possesses the ability to modify the ‘environment’ of the raw materials mixture so that the proteins present in the PC matrix can act as true emulsifiers. As mentioned above, the main role of ES is the ion exchange of calcium ions (of the insoluble calcium para-caseinate) for sodium ions, resulting in the development of the more soluble sodium para-caseinate [14,31,32]. However, less emulsified fat was observed in PC samples with KC addition, which presented the highest values of fat globule size. Furthermore, the difference value describes the difference between the smallest and the largest fat globule observed in the microphotographs of the investigated PC samples. When comparing individual samples, it was found that the fat globule size was statistically significantly different (*p* < 0.05). Furthermore, a significant difference (*p* < 0.05) was reported between the samples with the addition of PG and the samples with the remaining hydrocolloids applied (AG and KC). Thus, it can be stated that PC with AG and KG addition presented similar structures and sizes of fat globules. This similarity could probably be explained by the fact that both hydrocolloids are derived from seaweed [33]. Additionally, the size values of the fat globules for the samples prepared with PG were similar to those of CS. This phenomenon could be explained by the protein nature of gelatin [30]. The size of fat droplets could affect the consistency of PC; a greater number of small fat droplets might disorder the continuity of the protein matrix less intensively compared to the presence of a smaller number of orders of magnitude larger fat droplets, resulting in the development of a firmer structure [19].

**Figure 6:**
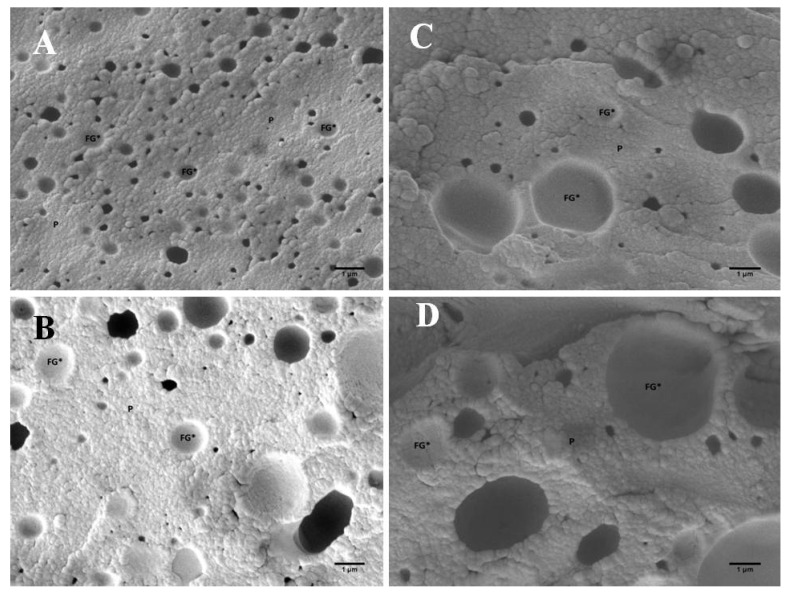
Scanning electron microscopy images of the model processed cheese (scale bars represent 1 μm; 10,000× magnification). (**A**) Sample with emulsifying salts; (**B**) sample with agar; (**C**) sample with κ-carrageenan; (**D**) sample with gelatin; FG*—void space of fat globules; P—protein structure.

In Figure 4, images of PC samples with hydrocolloid addition [AG (part A), KC (part B), PG (part C), and CS (part D)] are shown (at 5000 × magnification). Based on the size of the fat globules, it is evident (Figure 4) that the largest fat globules, or empty fat globule spaces, are in the AG-containing PC samples (Figure 4, part A). Furthermore, it is also possible to observe the association of the KC and casein fractions that form a compact protein-polysaccharide matrix (Figure 4, part B). A more detailed structure of the developed model PC samples is shown in Figure 5 and Figure 6. In particular, it can be seen that the samples produced with AG (Figure 5, part B, and Figure 6, part B) presented a less homogeneous PC matrix (heterogeneous structure compared to the microstructure of the CS with ES addition; Figure 5, part A, and Figure 6, part A). In addition, fat globules were not well noticeable in the CS at 1000× magnification (Figure 4D). Thus, the structure of the CS could still be characterized as homogeneous and uniform. PC containing larger fat globules has a smoother and softer consistency compared to PC with small fat globules [3,34].

## 4. Conclusions

The impact of three different hydrocolloids [agar (AG), κ-carrageenan (KC), or gelatin (PG)] on the viscoelastic properties and microstructure of PC manufactured without ES was evaluated during a storage time of 60 days (at 6 ± 2 °C). The application of hydrocolloids led to the manufacture of PC samples with divergent viscoelastic characteristics and microstructure. Furthermore, the addition of KC to PC samples resulted in increased values of G′, followed by those of AG. The control sample presented values of viscoelastic moduli similar to those of the PG sample. Moreover, a practical conclusion of the current study could be considered that for consumers who prefer PC products with softer consistency, the addition of PG could be recommended. On the other hand, if consumers prefer PC products with a firmer consistency, KC or AG could be applied. Last but not least, based on the results obtained from this study, it could be concluded that the application of AG, KC, or PG might lead to the development of new PC products with better health-promoting properties that meet the growing demands of consumers.

## Figures and Tables

**Table 1:** Formulation of the raw materials and processing parameters used for the processed cheese samples ***.

Raw Materials Processing Parameters	Composition % (*w/w*)			
	CS *	AG *	CR *	PG *
Raw materials				
Water	35.4	33.2	33.2	33.2
Edam cheese	43.1	47.7	47.7	47.7
Butter	19.0	18.1	18.1	18.1
Emulsifying salts **	2.5	none	none	none
Hydrocolloid	none	1.0	1.0	1.0
Processing parameters				
Stirring speed (rpm)	3000	3000	3000	3000
Holding time (s)	60	60	60	60
Target temperature (°C)	90	90	90	90
Total time (min)	11	11	11	11

* CS—sample with emulsifying salts; AG—sample with agar; * CR—sample with κ-carrageenan; * PG—sample with gelatin. ** Monosodium phosphate (NaH_2_PO_4_), disodium hydrogen phosphate (Na_2_HPO_4_), tetrasodium diphosphate (Na_4_P_2_O_7_), and sodium salt of polyphosphate having chain length n ≈ 20 were used in a ratio of 1.9:1.6:1.4:1.4. *** The total weight of the samples produced ranged from 1015.8 to 1026.2 g per batch. The weight of the sample in one container was approximately 90 ± 2 g.

**Table 2:** Values of the complex modulus of elasticity, complex viscosity, and loss tangent of the model processed cheese samples during a storage time of 60 days (at 6 ± 2 °C).

	Storage Time (days)											
	1			14			30			60		
Hydrocolloid	G* (Pa)	ŋ (Pa∙s)	tan δ	G* (Pa)	ŋ (Pa∙s)	tan δ	G* (Pa)	ŋ (Pa∙s)	tan δ	G* (Pa)	ŋ (Pa∙s)	tan δ
CS *	7445 ± 197 ^a,A^	1335 ± 2 ^a,A^	0.6 ^a,A^	8866 ± 213 ^a,B^	1257 ± 2 ^a,B^	0.6 ^a,A^	9441 ± 268 ^a,C^	1075 ± 3 ^a,C^	0.5 ^a,B^	10778 ± 314 ^a,D^	148 ± 1 ^a,D^	0.5 ^a,B^
AG *	44159 ± 576 ^b,A^	1624 ± 3 ^b,A^	0.2 ^b,A^	39817 ± 512 ^b,B^	2758 ± 3 ^b,B^	0.2 ^b,A^	38523 ± 451 ^b,B^	3437 ± 3 ^b,C^	0.2 ^b,A^	36950 ± 425 ^b,C^	4129 ± 3 ^b,D^	0.1 ^b,B^
CR *	85682 ± 931 ^c,A^	6286 ± 4 ^c,A^	0.2 ^b,A^	80343 ± 889 ^c,B^	7450 ± 3 ^c,B^	0.2 ^b,A^	77249 ± 817 ^c,C^	10692 ± 4 ^c,C^	0.2 ^b,A^	75521 ± 756 ^c,D^	12016 ± 4 ^c,D^	0.1 ^b,B^
PG *	10504 ± 278 ^d,A^	1011 ± 2 ^d,A^	0.3 ^c,A^	8090 ± 195 ^d,B^	1081 ± 2 ^d,B^	0.4 ^c,B^	7687 ± 187 ^d,B^	1242 ± 2 ^d,C^	0.4 ^c,B^	5369 ± 138 ^d,C^	2016 ± 2 ^d,D^	0.5 ^c,C^

* CS—control sample with 2.5% (*w*/*w*) emulsifying salts; * AG—sample with 1% (*w*/*w*) agar; * CR—sample with 1% (*w*/*w*) κ-carrageenan; * PG—sample with 1% (*w*/*w*) gelatin. Means within a column (the difference between hydrocolloid type; comparing the control sample was also included) followed by different superscript letters differ (*p* < 0.05). Means within a row (the difference between storage time; comparing the same hydrocolloid type; the control sample was also included) followed by different uppercase letters differ (*p* < 0.05). G*, ŋ, and tan δ were evaluated individually.

**Table 3:** Size (diameter) of fat globules of the model processed cheese samples; the results are expressed as mean ± standard deviation.

	Size of Fat Globules			
Hydrocolloid	Mean (μm)	Minimum Size (μm)	Maximum Size (μm)	Difference (μm)
CS *	0.199 ± 0.015 ^a^	0.029 ± 0.034 ^a^	0.790 ± 0.052 ^a^	0.761 ± 0.018 ^a^
AG *	0.756 ± 0.084 ^b^	0.135 ± 0.071 ^b^	4.583 ± 0.098 ^b^	4.448 ± 0.027 ^b^
CR *	0.761 ± 0.056 ^b^	0.145 ± 0.028 ^b^	2.507 ± 0.067 ^c^	2.362 ± 0.039 ^c^
PG *	0.490 ± 0.062 ^c^	0.052 ± 0.049 ^c^	3.890 ± 0.073 ^d^	3.838 ± 0.024 ^d^

* CS—control sample with emulsifying salts; * AG—sample with agar; * CR—sample with κ-carrageenan; * PG—sample with gelatin. Means within a column (the difference between hydrocolloid type; comparing the control sample was also included) followed by different superscript letters differ (*p* < 0.05).

## Data Availability

The data presented in this study are available on request from the corresponding author.
